# Neurological manifestations in metabolic dysfunction syndrome are associated with cumulative metabolic burden beyond hyperglycaemia: a translational study

**DOI:** 10.3389/fendo.2026.1946996

**Published:** 2026-09-28

**Authors:** Jinfang Ma, Yi Tang, Qingxing Xie, Shishi Xu, Sumin Shen, Yuwei Zhang, Fang Zhang, Jing Li, Hui Jiang, Xiaodan Zhong, Nanwei Tong

**Affiliations:** 1 Department of Endocrinology and Metabolism, West China Hospital, Sichuan University, Chengdu, China; 2 Laboratory of Diabetes and Metabolism Research, West China Hospital, Sichuan University, Chengdu, China; 3 Department of Endocrinology, Chengdu Fifth People’s Hospital, Chengdu, China; 4 Department of Endocrinology, Qionglai People’s Hospital, Chengdu, China; 5 Department of Clinical Research Management, West China Hospital, Sichuan University, Chengdu, China

**Keywords:** autonomic dysfunction, hyperglycaemia, metabolic syndrome, neuropathy, peripheral nerve damage

## Abstract

**Objective:**

To determine whether neurological injury in metabolic syndrome (MetS) extends beyond hyperglycaemia-driven neuropathy.

**Methods:**

We employed a translational design combining controlled rat models of isolated and combined MetS components with a community-based cross-sectional human study. Preclinically, ten rat models (n = 6 per group) representing various MetS phenotypes were assessed for metabolic parameters and peripheral neurological function. Clinically, 1,176 adults were stratified by glycaemic and MetS status according to International Diabetes Federation (IDF) criteria. Participants underwent anthropometric measurements, fasting biochemistry, peripheral nerve function assessment by electromyography (EMG), and autonomic function evaluation by heart rate variability (HRV) and electrogastrography (EGG).

**Results:**

In animal models, hindpaw withdrawal latencies were prolonged and nerve conduction velocity (NCV) was significantly reduced across multiple MetS phenotypes, irrespective of glycaemic status. In humans, the MetS with euglycaemia group exhibited peripheral neuropathy, with reduced nerve amplitudes and slowed NCV (P < 0.05). HRV analysis revealed significant cardiac autonomic dysfunction in the MetS with euglycaemia group, including reductions in the root mean square of successive differences (RMSSD), the standard deviation of normal-to-normal intervals (SDNN), total power (TP), high-frequency (HF) power, and the Poincaré long-axis index (SD2) (all P < 0.05)—deficits that were comparable to or exceeded those in the isolated hyperglycaemia group. Gastrointestinal dysautonomia, however, was prominent only in the MetS with hyperglycaemia subgroup.

**Interpretation:**

Both peripheral and autonomic neuropathies in MetS are associated with cumulative metabolic burden rather than hyperglycaemia alone, with organ-specific susceptibility patterns. These findings challenge a purely glucocentric view of MetS-related neural injury and support comprehensive risk-factor management in all MetS patients, irrespective of glycaemic status.

## 1 Introduction

Metabolic syndrome (MetS) constitutes a cluster of interrelated metabolic disturbances—central obesity, hypertension, dyslipidaemia, and hyperglycaemia—that collectively amplify the risk of cardiovascular disease (CVD), type 2 diabetes (T2D), chronic kidney disease (CKD), and systemic end-organ damage (1–3). Affecting approximately one quarter of the world’s adult population—over one billion people—MetS represents a critical public health burden, driven largely by rising obesity rates and sedentary lifestyles (1, 2, 4). The underlying pathophysiology involves insulin resistance (IR), chronic low-grade inflammation, oxidative stress, and endothelial dysfunction, which synergistically promote microvascular and macrovascular complications (5, 6). Whilst substantial research has elucidated MetS-associated damage to cardiovascular, renal, and retinal systems, neurological manifestations beyond hyperglycaemia-induced neuropathies remain profoundly understudied.

A growing body of epidemiological evidence has established associations between MetS and peripheral neuropathy beyond the context of diabetes. Callaghan and colleagues demonstrated that diabetes and obesity are the main metabolic drivers of peripheral neuropathy in a large population-based cohorta (7, 8), and further showed that central obesity is independently associated with neuropathy in severely obese individuals (9, 10). Ziegler et al., in the MONICA/KORA Augsburg Surveys, reported that the prevalence of polyneuropathy in prediabetes and diabetes is significantly associated with abdominal obesity and macroangiopathy (11). These landmark studies collectively suggest that non-glycaemic components of MetS — particularly obesity and dyslipidaemia — contribute to neural injury independently of hyperglycaemia. However, most prior investigations have focused on peripheral neuropathy in diabetic or pre-diabetic populations, leaving the neurological phenotype in euglycaemic individuals with full MetS inadequately characterised. Moreover, systematic evaluation of both peripheral and autonomic nerve function across the full spectrum of MetS components — from isolated hyperglycaemia to multi-component MetS with and without hyperglycaemia — remains limited.

Conventionally, peripheral and autonomic neuropathies have been attributed predominantly to hyperglycaemia in diabetic populations (12, 13). However, emerging evidence suggests that non-glycaemic components of MetS independently contribute to neural injury. Dyslipidaemia may induce oxidative stress and mitochondrial dysfunction in peripheral nerves (14); hypertension can accelerate microvascular ischaemia within the vasa nervorum (15); and visceral adiposity promotes pro-inflammatory cytokines that disrupt the blood–nerve barrier (16). Notably, studies indicate that sympathetic neuropathy in metabolic organs such as the liver manifests during pre-diabetic states, mediated by TNFα-driven neuro-immune dysregulation rather than hyperglycaemia alone (17). Despite these insights, neurological impairments in euglycaemic MetS populations remain inadequately characterised. Most clinical investigations have prioritised hyperglycaemia as the primary neurotoxic driver (18, 19), overlooking potential contributions from isolated hypertension, obesity, or dyslipidaemia. This knowledge gap impedes early intervention for MetS patients who develop neural complications prior to a diabetes diagnosis.

To address this critical gap, we employed a translational approach combining controlled animal models of MetS—and its component phenotypes—with a nested community-based human study. Rat models enabled systematic observation of early neural damage across the spectrum of metabolic disturbances. In the human cohort, participants were stratified into four groups: normoglycaemic controls, isolated hyperglycaemia, MetS with euglycaemia, and MetS with hyperglycaemia. All participants underwent standardised metabolic profiling and comprehensive neural assessments. This study aims to test the hypothesis that neurological injury in MetS is not exclusive to hyperglycaemia, but rather arises from synergistic interactions amongst its individual components.

## 2 Methods

### 2.1 Animal experiments

#### 2.1.1 Rats

Male 4-week-old Wistar and spontaneously hypertensive rats (SHR) of the Wistar strain (from Vital River Laboratory Animal Technology Co., Ltd, Beijing) were housed at 24 ± 1 °C with a 12-h light/dark cycle and ad libitum access to water. After one week of adaptive feeding, Wistar rats were individually fed with control diet (1010009)/high-fat, high-fructose, and high-cholesterol diet (XT301)/45% high-fat diet (XTHF45) for a duration of 28 weeks to develop normal (N), hyperlipidemia (L), and obesity (O) models. Similarly, SHR rats were given control diet/0.5% sodium cholate diet (XT109C)/45% high-fat diet to construct hypertension (H), hypertension + hyperlipidemia (HL), and hypertension + obesity + hyperlipidemia (MetS2: HOL) models. The diet-induced obesity model (45% high-fat diet) was established according to published high-fat diet protocols in rats (20, 21); the high-fat diet plus low-dose streptozotocin protocol used to model T2D followed the established method of Srinivasan et al. (22); the diet-induced hyperlipidaemia models were based on established high-fat–high-cholesterol and metabolic syndrome diet models (23, 24); and the SHR model of genetic hypertension followed standard protocols (25). Detailed diet compositions for each rat model are summarised in **Supplementary Table S1**.

A subset of Wistar rats on the 45% high-fat diet received two intraperitoneal injections of 1% STZ after 8 weeks to induce T2D, forming a T2D+obesity (DO) model. These were then fed an atherosclerosis-inducing diet to create a T2D+obesity+hyperlipidemia (MetS1: DOL) model. Similarly, T2D was induced in SHR rats to establish T2D+hypertension+obesity (MetS3: DHO) or T2D+hypertension+obesity+hyperlipidemia (MetS4: DHOL) models.

In total, the rats were randomly assigned into ten groups, each consisting of 6 animals. Rats were kept on diet intervention for 28 weeks. All feeds were purchased from Jiangsu Synergetic Bioengineering Co., Ltd.

Body weight and blood pressure were measured every 2–4 weeks. All animal procedures were conducted in accordance with the National Institutes of Health Guidelines and were approved by the Institutional Animal Care and Use Committee (IACUC) at Sichuan University.

#### 2.1.2 *In vivo* biochemical measurements

Serum insulin, triglyceride (TG), total cholesterol (TC), low-density lipoprotein cholesterol (LDL-C) and high-density lipoprotein cholesterol (HDL-C) were measured using commercial assay kits (Elabscience). Glucose homeostasis was characterised by fasting glucose (Accu-Chek Performa), fasting insulin (Cloud-Clone), and HOMA-IR.

#### 2.1.3 Nerve function assessment

The thermal sensitivity of the tibial nerve was evaluated using a RB200 Intelligent Hot Plate, and the conduction velocity of the sciatic nerve was measured utilizing the BL-420F Biological Function Experiment System.

### 2.2 The cross-sectional community-based study

#### 2.2.1 Study population

All participants were recruited from a screening survey conducted in Mianzhu City and the Longquan and Pidu area of Chengdu City, Sichuan province of China, between December 2020 and February 2022, from the Glucose Metabolism Cohort Study and the Natural Population Cohort Study at West China Hospital of Sichuan University (registration number: ChiCTR2100044819 and ChiCTR1900024623). This study’s inclusion criteria include: (1) being 20 years old or above, and (2) having either a local household registration or having lived in the local area for a minimum of six months. The exclusion criterion pertains to individuals who intend to relocate or depart from the area within the upcoming two-year period. Information about lifestyle and medications were obtained by questionnaire.

In this study, the diagnosis of MetS was primarily based on the diagnostic criteria jointly released in 2009 by international organizations such as the International Diabetes Federation. A subject was considered to have MetS if they met three or more of the following criteria:

1. Central obesity: waist circumference >90 cm for males and >80 cm for females (26).
2. Elevated triglycerides: Serum TG levels were ≥ 1.7 mmol/L (or the subject was receiving corresponding treatment).
3. Reduced HDL-C: Levels were < 1.0 mmol/L for males and < 1.3 mmol/L for females (or the subject was under corresponding treatment).
4. Hypertension: Systolic blood pressure was ≥ 130 mmHg or diastolic blood pressure was ≥ 85 mmHg (or the subject was receiving corresponding treatment).
5. Elevated fasting glucose: Levels were ≥ 5.6 mmol/L (or the subject was undergoing corresponding treatment or had previously been diagnosed with T2D).

Participants were divided into four groups: (i) normoglycaemic controls- no hyperglycaemia and fewer than three MetS criteria; (ii) hyperglycaemia alone -hyperglycaemia but fewer than three MetS criteria; (iii) MetS with euglycaemia- at least three MetS criteria with normoglycaemia; and (iv) MetS with hyperglycaemia - at least three MetS criteria together with hyperglycaemia.

#### 2.2.2 Anthropometric measurements

Individual height, weight, waist circumference (WC) and blood pressure were measured by trained physicians.

#### 2.2.3 Biochemical measurements

Blood samples were collected after a 12h overnight fasting for laboratory tests, including fasting glucose and lipid profile (TG, HDL-C).

#### 2.2.4 Electromyography examination

In the electromyography (EMG) evaluation of our study population, motor nerve conduction (right peroneal and tibial nerves), sensory nerve conduction (right superficial peroneal nerve), and sympathetic skin responses (right median nerve) were assessed using usual techniques (27, 28), allowing comprehensive evaluation of both motor and sensory peripheral nerve function.

#### 2.2.5 Electrocardiogram examination and heart rate variability analysis

The ECG was acquired over a period of at least 5 minutes during resting conditions to ensure adequate capture of baseline cardiac activity. Subsequently, heart rate variability (HRV) analysis was performed using Kubios HRV Premium software (Version 3.4) with established standards (29, 30).

#### 2.2.6 Electrogastrogram examination

In our electrogastrography (EGG) study, participants fasted and underwent recording in a resting state. As described in previously established protocols (31, 32), bipolar cutaneous electrodes were placed over the gastric antrum and corpus. Postprandial recordings were also obtained as needed.

#### 2.2.7 Ethics

The human study was approved by the Biomedical Ethics Committee of West China Hospital, Sichuan University (Ref: 2020-264), and all participants provided written informed consent.

### 2.3 Statistical analysis

Data are presented as mean ± standard deviation (SD). Analyses were performed using GraphPad Prism 8.0 (San Diego, CA, USA). Normally distributed data were compared using unpaired t-tests (two groups) or one-way ANOVA with Bonferroni’s *post hoc* test (≥3 groups). Non-normally distributed data were compared using Mann–Whitney U tests (two groups) or Kruskal–Wallis tests with Dunn’s *post hoc* correction (≥3 groups). Normality was assessed using the Shapiro–Wilk test. Bonferroni correction was applied within each individual ANOVA for multiple comparisons; global correction across all endpoint families was not performed, given the exploratory nature of the multi-modal assessment. P < 0.05 was considered significant.

## 3 Results

### 3.1 Peripheral neurological impairments in rat models of MetS are associated with cumulative metabolic dysregulation rather than isolated hyperglycaemia

To validate the metabolic phenotypes of the established rat models, we first assessed body weight, blood pressure, lipid profiles, and glucose homeostasis. Subsequently, to evaluate peripheral nerve function, we measured hindpaw withdrawal latencies (HWLs) as an indicator of sensory nociceptive function, and sciatic nerve conduction velocity (SNCV) as a measure of motor/mixed nerve function.

Rats fed a high-fat diet (HFD) exhibited a >20% increase in body weight relative to controls by week 12 (P < 0.05), confirming successful obesity induction. In contrast, rats on combined high-fat, high-fructose, and high-cholesterol diets—with concurrent hypertension or hyperglycaemia—showed attenuated weight gain or even transient body weight loss (**Figure 1A**). Spontaneously hypertensive rats (SHR) developed systolic blood pressure exceeding 140 mmHg by week 6, which peaked between weeks 16 and 28 (**Figure 1B**). Dyslipidaemic models displayed elevated triglycerides (TG), total cholesterol (TC), low-density lipoprotein cholesterol (LDL-C), and reduced high-density lipoprotein cholesterol (HDL-C) at 20 weeks, indicating robust diet-induced dyslipidaemia (**Figure 1C**). Aortic atherosclerosis was directly assessed in MetS model rats, confirming that dyslipidemic groups successfully developed atherosclerotic lesions, whilst single-component or non-dyslipidemic groups did not (**Supplementary Figure 1**).

**Figure 1:**
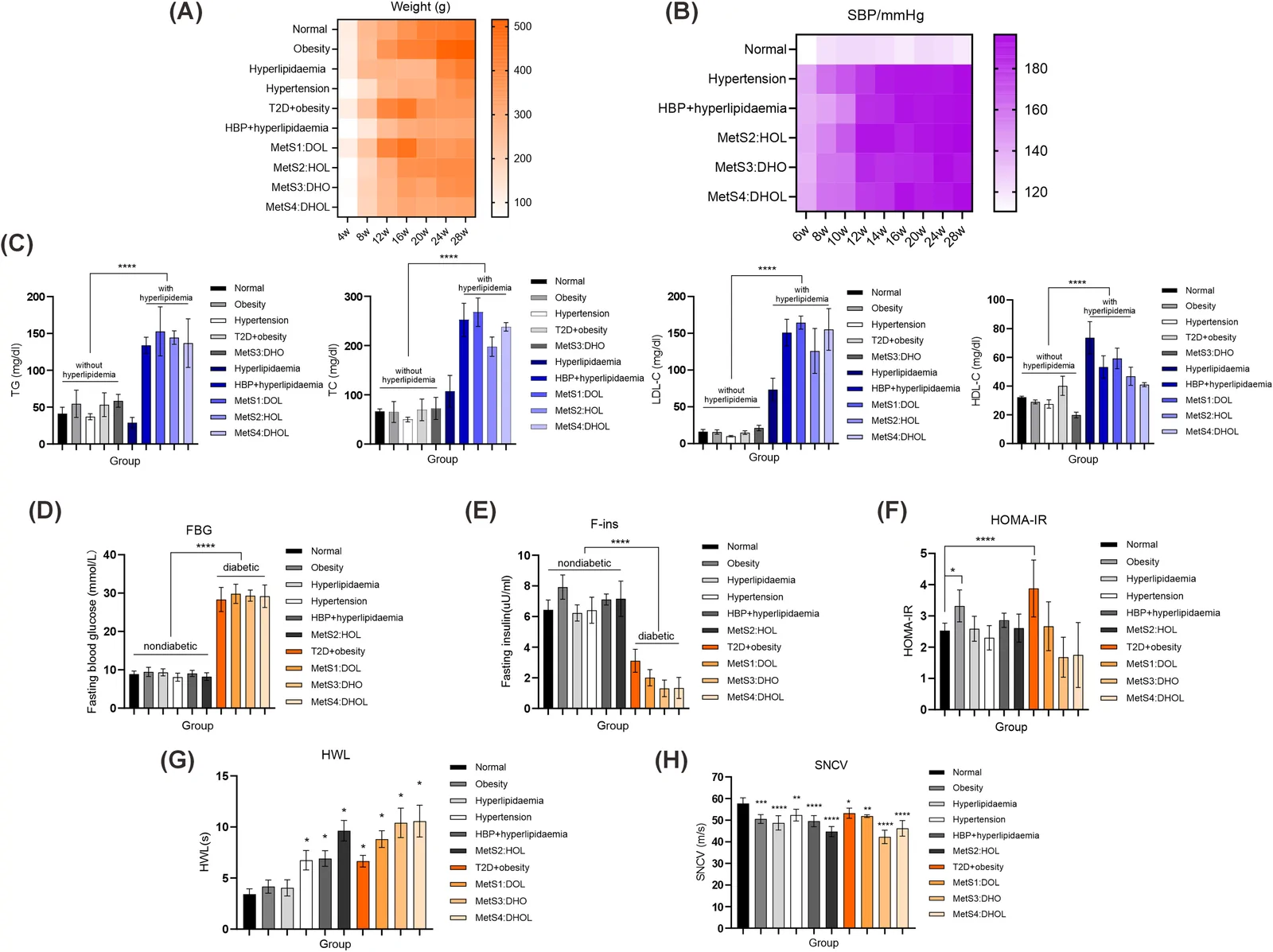
General metabolic characteristics and peripheral neurological assessment of various MetS models and corresponding control groups. **(A)** Body weight variations over time. **(B)** Systolic blood pressure (SBP) in each group. **(C)** Serum levels of triglycerides (TG), total cholesterol (TC), low-density lipoprotein cholesterol (LDL-C), and high-density lipoprotein cholesterol (HDL-C). **(D)** Fasting blood glucose (FBG, mmol/L). **(E)** Serum fasting insulin (F-ins, uU/ml). **(F)** HOMA-IR values. **(G)** Hindpaw withdrawal latency (HWL, seconds). **(H)** Sciatic nerve conduction velocity (NCV, m/s). n = 6 rats per group. MetS, metabolic syndrome; T2D+obesity, type 2 diabetes + obesity; HBP+hyperlipidaemia, hypertension + hyperlipidaemia; MetS1:DOL, type 2 diabetes + obesity + hyperlipidaemia; MetS2:HOL, hypertension + obesity + hyperlipidaemia; MetS3:DHO, type 2 diabetes + hypertension + obesity; MetS4:DHOL, type 2 diabetes + hypertension + obesity + hyperlipidaemia. Values are presented as mean ± SD. Statistical comparisons amongst multiple groups were performed using one-way ANOVA with Bonferroni’s *post hoc* test, and comparisons between two groups were performed using unpaired t-tests. *P < 0.05, **P < 0.01, ***P < 0.001, ****P < 0.0001.

Streptozotocin (STZ)-treated rats maintained fasting glucose levels >16.7 mmol/L, confirming stable T2D induction (**Figure 1D**). Fasting insulin levels were significantly reduced in T2D models, particularly in multi-component MetS groups (**Figure 1E**). In obese rats, HOMA-IR analysis revealed elevated fasting insulin and higher HOMA-IR values (P < 0.05), despite normal fasting glucose, indicative of prominent insulin resistance (IR). Notably, although the T2D+obesity group showed reduced insulin levels, HOMA-IR remained significantly elevated (3.88 ± 0.37 vs. 2.53 ± 0.09 in controls, P < 0.05), suggesting that IR predominates in this phenotype, consistent with human obese T2D. In multi-component MetS rats, the metabolic profile transitioned from IR towards predominant insulin deficiency, implying accelerated β-cell dysfunction (**Figure 1F**). Collectively, these data confirm the successful establishment of hypertension, hyperlipidaemia, and hyperglycaemia in the respective rat models.

To evaluate terminal sensory function of the tibial nerve, we measured HWLs in response to heat plate stimulation on the plantar surfaces of both hind paws. As the tibial nerve provides major sensory innervation to the plantar region, HWLs serve as a reliable indicator of distal tibial nerve sensory function. The mean HWL in the control group was 3.42 ± 0.21 seconds. In contrast, HWLs were significantly prolonged in the T2D and hypertension groups (**Figure 1G**), indicating impaired superficial pain perception. Notably, rats with combined T2D and hypertension—or with an increasing number of MetS components—exhibited a more pronounced attenuation of pain sensitivity.

We next assessed SNCV. The average SNCV in the normal control group was 57.74 ± 1.05 m/s. Compared with controls, SNCV was significantly reduced across all MetS model groups and their corresponding controls (**Figure 1H**). As SNCV reflects motor/mixed nerve function, these findings demonstrate that MetS-related peripheral nerve impairments affect both sensory and motor modalities. Importantly, the observed functional deficits occurred irrespective of glycaemic status, suggesting that peripheral nerve injury in MetS is associated with cumulative metabolic burden rather than hyperglycaemia alone.

The metabolic and neuropathic phenotypes of the ten rat models are summarised in **Supplementary Table S2**.

### 3.2 Peripheral neuropathic alterations in the community-based population are associated with cumulative metabolic components beyond hyperglycaemia

To further validate the findings from the animal experiments, we conducted epidemiological investigations and neuropathy assessments in a human cohort. Amongst participants in the metabolic syndrome screening survey, 1,176 individuals underwent electromyography (EMG). Based on the study design, participants were stratified into four groups: normoglycaemic controls, hyperglycaemia alone, MetS with euglycaemia (MetS+euglycaemia), and MetS with hyperglycaemia (MetS+hyperglycaemia). The clinical and biochemical characteristics of these participants are summarised in **Table 1**.

**Table 1:** Characteristics of the study population that underwent EMG.

Variables	Euglycaemia (n=633)	Hyperglycaemia (n=158)	MetS+euglycaemia (n=117)	MetS+hyperglycaemia (n=268)
Age (Years)	55.69 ± 0.39	56.25 ± 0.72	54.36 ± 0.82	57.74 ± 0.52
Sex (Male/Female)	139/494	60/98	46/71	91/177
BMI (kg/m^2^)	23.76 ± 0.12	23.42 ± 0.21	26.32 ± 0.29*	26.35 ± 0.21*
WC (cm)	79.92 ± 0.34	79.15 ± 0.55	86.87 ± 0.77*	88.66 ± 0.45*
WcHtR	0.51 ± 0.002	0.50 ± 0.003	0.56 ± 0.005*	0.56 ± 0.003*
TG (mmol/L)	1.41 ± 0.036	1.35 ± 0.085	2.58 ± 0.16*	2.53 ± 0.14*
FPG (mmol/L)	4.87 ± 0.016	6.93 ± 0.15*	4.97 ± 0.03	7.01 ± 0.11*
HDL-C (mmol/L)	1.80 ± 0.019	1.95 ± 0.042	1.36 ± 0.035*	1.60 ± 0.027*
SBP (mmHg)	124.50 ± 0.71	126.40 ± 1.28	134.70 ± 1.39*	138.40 ± 1.04*
DBP (mmHg)	78.32 ± 0.38	76.93 ± 0.71	83.88 ± 0.84*	83.08 ± 0.59*

Data are expressed as mean ± SEM. BMI, body mass index, WC waist circumference, WcHtR Waist Circumference-to-Height Ratio, TG triglycerides, FPG, fasting plasma glucose, HDL-C, high-density lipoprotein cholesterol, SBP, systolic blood pressure, DBP, diastolic blood pressure. Comparisons amongst the four groups were performed using one-way ANOVA with Bonferroni’s *post hoc* test. *Denotes P < 0.05 vs. the euglycaemia group.

Motor conduction velocity (MCV), sensory conduction velocity (SCV), and sympathetic skin response (SSR) were systematically assessed across the four groups.

For the right peroneal nerve, the hyperglycaemia and MetS+hyperglycaemia groups exhibited significantly reduced amplitudes at the ankle segment and the fibula-to-ankle segment compared with normoglycaemic controls (both P < 0.001), whereas no significant differences were observed between the MetS+euglycaemia group and controls (**Figure 2A**). Likewise, conduction velocities of the right peroneal nerve (fibula-to-ankle segment) were markedly slower in the hyperglycaemia and MetS+hyperglycaemia groups (P < 0.001), but the MetS+euglycaemia group did not differ significantly from controls (**Figure 2A**).

**Figure 2:**
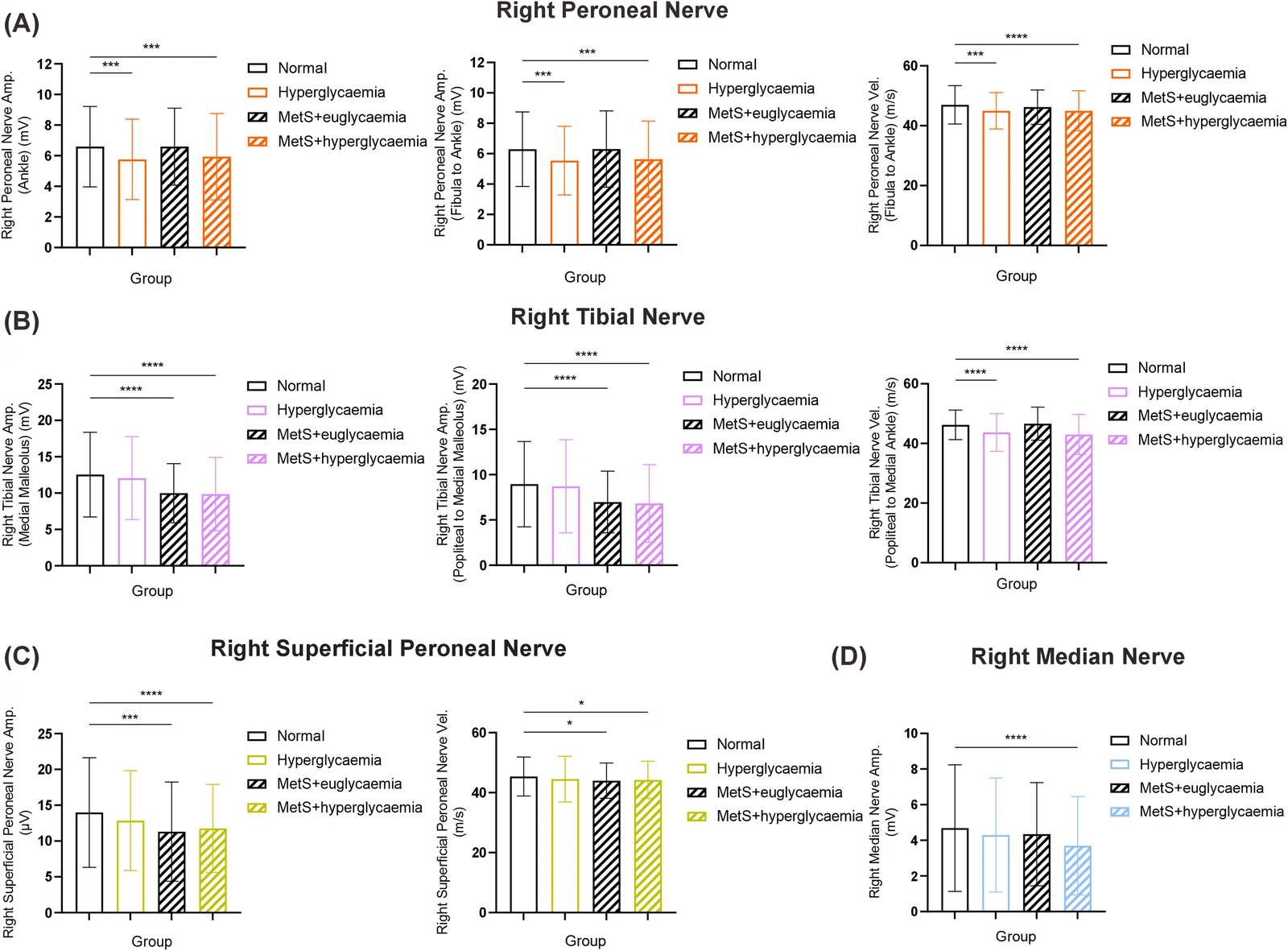
Peripheral nerve impairment detected by electromyography (EMG) in the community-based population. **(A)** Ankle segment amplitude, fibula-to-ankle segment amplitude, and conduction velocity of the right peroneal nerve. **(B)** Medial malleolar segment amplitude, popliteal-to-medial malleolus segment amplitude, and conduction velocity of the right tibial nerve. **(C)** Amplitude and conduction velocity of the right superficial peroneal nerve. **(D)** Amplitude of the right median nerve. Amp, amplitude; Vel, velocity. Values are presented as mean ± SD. Statistical comparisons amongst the four groups were performed using one-way ANOVA with Bonferroni’s *post hoc* test. *P<0.05, ***P<0.001, ****P<0.0001.

For the right tibial nerve, both medial malleolar amplitude and popliteal-to-medial malleolus segment amplitude were significantly attenuated in the MetS+euglycaemia and MetS+hyperglycaemia groups compared with controls (both P < 0.0001, **Figure 2B**). Although the hyperglycaemia group showed a numerical decline in these amplitudes, the differences did not reach statistical significance (**Figure 2B**). Notably, conduction velocities of the right tibial nerve (popliteal-to-medial malleolus segment) were significantly reduced in both the hyperglycaemia and MetS+hyperglycaemia groups (P < 0.0001, **Figure 2B**).

In sensory nerve assessments, the right superficial peroneal nerve showed significant reductions in both amplitude and conduction velocity in the MetS+euglycaemia and MetS+hyperglycaemia groups relative to controls (**Figure 2C**). Although the hyperglycaemia group exhibited a downward trend in these parameters, statistical significance was not achieved (**Figure 2C**).

Regarding SSR, the amplitude of the right median nerve showed a decreasing trend in the hyperglycaemia and MetS+euglycaemia groups; however, only the MetS+hyperglycaemia group demonstrated a statistically significant reduction compared with controls (P < 0.0001, **Figure 2D**).

Collectively, these EMG findings suggest that whilst hyperglycaemia contributes substantially to motor nerve dysfunction, the presence of additional MetS components—particularly in the absence of hyperglycaemia—is associated with tibial and superficial peroneal sensory nerve impairments. This differential pattern underscores the contributory role of metabolic components beyond hyperglycaemia in peripheral neuropathy.

### 3.3 Autonomic nerve dysfunction in the community-based population is associated with cumulative metabolic burden

#### 3.3.1 Heart rate variability

Amongst participants who underwent EMG, 731 also underwent electrocardiography (ECG), and HRV analysis was performed using Kubios HRV Premium software. Participants were stratified into the same four groups as above. Clinical characteristics are summarised in **Table 2**.

**Table 2:** Characteristics of the study population that underwent ECG.

Variables	Euglycaemia (n=395)	Hyperglycaemia (n=81)	MetS+euglycaemia (n=87)	MetS+hypergycaemia (n=168)
Age (years)	55.04 ± 0.48	58.95 ± 1.01	55.06 ± 0.93	59.24 ± 0.64
Sex (Male/Female)	87/308	27/54	19/68	57/111
BMI (kg/m^2^)	23.55 ± 0.15	23.08 ± 0.29	26.11 ± 0.34*	26.49 ± 0.28*
WC (cm)	79.23 ± 0.42	78.21 ± 0.75	86.92 ± 0.91*	88.50 ± 0.59*
WcHtR	0.51 ± 0.003	0.50 ± 0.004	0.56 ± 0.006*	0.57 ± 0.004*
TG (mmol/L)	1.43 ± 0.05	1.32 ± 0.15	2.44 ± 0.17*	2.68 ± 0.21*
FPG (mmol/L)	4.80 ± 0.02	6.90 ± 0.23*	4.93 ± 0.04	7.00 ± 0.14*
HDL-C (mmol/L)	1.66 ± 0.02	1.85 ± 0.05*	1.30 ± 0.03*	1.54 ± 0.04*
SBP (mmHg)	123.20 ± 0.94	125.60 ± 1.54	133.50 ± 1.69*	138.00 ± 1.33*
DBP (mmHg)	78.37 ± 0.49	75.99 ± 0.88	83.12 ± 0.89*	83.42 ± 0.75*

Data are expressed as mean ± SEM. Comparisons amongst the four groups were performed using one-way ANOVA with Bonferroni’s *post hoc* test. *Denotes P < 0.05 compared to the euglycaemia group.

HRV parameters—including time-domain, frequency-domain, and nonlinear indices—were evaluated.

In time-domain analysis, RMSSD was significantly reduced in the hyperglycaemia, MetS+euglycaemia, and MetS+hyperglycaemia groups compared with controls (all P < 0.05), with the lowest values observed in the MetS+hyperglycaemia group (P < 0.0001, **Figure 3A**), suggesting a synergistic effect of metabolic components. Similarly, SDNN was significantly attenuated in these three groups relative to controls (P < 0.05 for hyperglycaemia; P < 0.0001 for MetS+euglycaemia and MetS+hyperglycaemia; **Figure 3A**).

**Figure 3:**
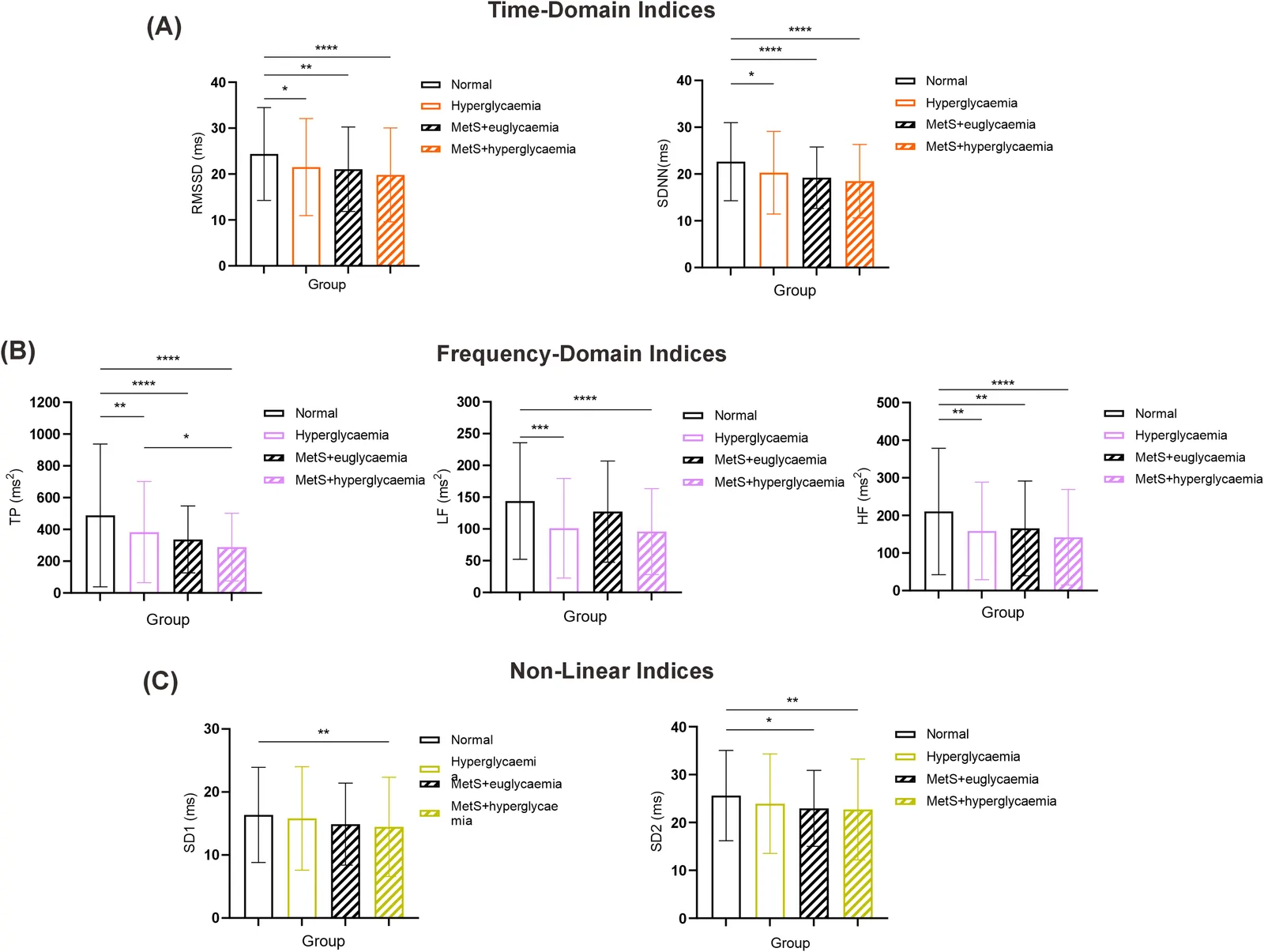
Heart rate variability (HRV) parameters in the community-based population. HRV metrics were compared across four metabolic groups: normal, hyperglycaemia, metabolic syndrome with euglycaemia (MetS+euglycaemia), and metabolic syndrome with hyperglycaemia (MetS+hyperglycaemia). A.Time-domain indices (RMSSD and SDNN). **(B)** Frequency-domain indices (TP, LF, and HF). **(C)** Non-linear indices (SD1 and SD2). Values are presented as mean ± SD. Statistical comparisons amongst the four groups were performed using one-way ANOVA with Bonferroni’s *post hoc* test. *P<0.05, **P<0.01, ***P<0.001, ****P<0.0001.

Frequency-domain analysis revealed a marked reduction in total power (TP) in the hyperglycaemia, MetS+euglycaemia, and MetS+hyperglycaemia groups compared with controls, with the MetS+hyperglycaemia group exhibiting the lowest TP values (vs. hyperglycaemia: P < 0.05; **Figure 3B**). Low-frequency (LF) power, reflecting sympathetic modulation, was significantly decreased in the hyperglycaemia and MetS+hyperglycaemia groups, whilst the MetS+euglycaemia group showed a non-significant downward trend (**Figure 3B**). High-frequency (HF) power, indicative of parasympathetic activity, was also significantly reduced across all three groups (hyperglycaemia and MetS+euglycaemia: P < 0.01; MetS+hyperglycaemia: P < 0.001; **Figure 3B**).

Nonlinear analysis demonstrated that SD1 (short-term variability) was significantly lower only in the MetS+hyperglycaemia group compared with controls, despite numerical declines in the hyperglycaemia and MetS+euglycaemia groups (**Figure 3C**). SD2 (long-term variability) showed significant reductions in both the MetS+euglycaemia and MetS+hyperglycaemia groups, whereas the hyperglycaemia group displayed a non-significant decrease (**Figure 3C**).

Collectively, these HRV findings indicate impaired cardiac autonomic function—characterised by reduced sympathetic and parasympathetic modulation—in all three affected groups. Importantly, the pronounced reductions observed in the MetS+euglycaemia group suggest that metabolic components other than hyperglycaemia independently contribute to cardiac autonomic dysfunction.

#### 3.3.2 Electrogastrography testing

Amongst participants who underwent EMG, 285 also underwent EGG. Participants were stratified into the same four groups, with clinical characteristics summarised in **Table 3**.

**Table 3:** Characteristics of the study population that underwent EGG.

Variables	Euglycaemia (n=166)	Hyperglycaemia (n=39)	MetS+euglycaemia (n=25)	MetS+hypergycaemia (n=55)
Age (Years)	55.14 ± 0.41	56.38 ± 0.79	54.00 ± 1.24	56.29 ± 0.82
Sex (Male/Female)	36/130	16/23	7/18	18/37
BMI (kg/m^2^)	23.60 ± 0.23	23.93 ± 0.48	25.97 ± 0.64*	26.77 ± 0.37*
WC (cm)	80.10 ± 0.69	81.05 ± 1.34	85.72 ± 1.77*	89.71 ± 1.03*
WcHtR	0.51 ± 0.004	0.51 ± 0.009	0.55 ± 0.011*	0.568 ± 0.006*
TG (mmol/L)	1.33 ± 0.047	1.31 ± 0.10	2.31 ± 0.14*	2.58 ± 0.28*
FPG (mmol/L)	4.98 ± 0.03	6.94 ± 0.25	5.14 ± 0.07	6.84 ± 0.16*
HDL-C (mmol/L)	2.01 ± 0.04	2.02 ± 0.08	1.55 ± 0.09*	1.63 ± 0.06*
SBP (mmHg)	125.80 ± 1.29	126.60 ± 2.01	139.70 ± 2.34*	142.10 ± 2.29*
DBP (mmHg)	78.84 ± 0.71	75.66 ± 1.37	83.95 ± 2.13*	84.18 ± 1.16*

Data are expressed as mean ± SEM. Comparisons amongst the four groups were performed using one-way ANOVA with Bonferroni’s *post hoc* test. *Denotes P < 0.05 compared to the euglycaemia group.

EGG analysis revealed that the MetS+hyperglycaemia group exhibited significantly elevated percentages of gastric electrical rhythm disturbances (GERD) at the corpus during both fasting and postprandial periods compared with controls (both P < 0.05, **Figure 4A**). At the antrum, the MetS+hyperglycaemia group showed a substantial increase in postprandial gastric dysrhythmias (P < 0.001, **Figure 4A**). In contrast, the hyperglycaemia and MetS+euglycaemia groups showed trends towards increased dysrhythmias, but these did not reach statistical significance (**Figure 4A**).

**Figure 4:**
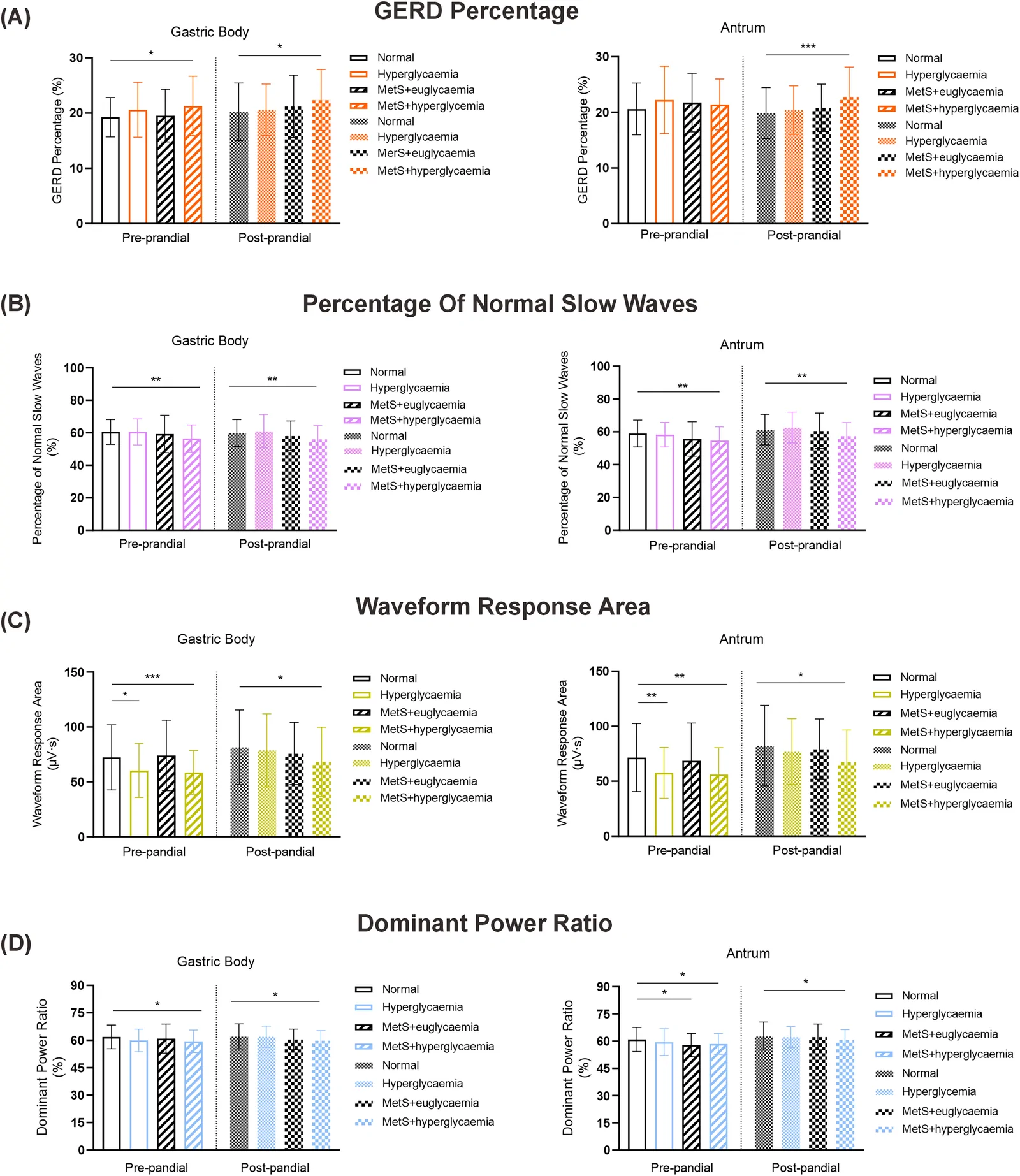
Electrogastrography (EGG) parameters in the community-based population. EGG metrics were compared across four metabolic groups: normal, hyperglycaemia, metabolic syndrome with euglycaemia (MetS+euglycaemia), and metabolic syndrome with hyperglycaemia (MetS+hyperglycaemia). **(A)** Gastric Electrical Rhythm Disturbance (GERD) percentage at the gastric body and antrum during preprandial and postprandial recordings. **(B)** Percentage of normal slow waves at the gastric body and antrum during preprandial and postprandial recordings. **(C)** Waveform response area at the gastric body and antrum during preprandial and postprandial recordings. **(D)** Dominant power ratio at the gastric body and antrum during preprandial and postprandial recordings. Values are presented as mean ± SD. Statistical comparisons amongst the four groups were performed using one-way ANOVA with Bonferroni’s *post hoc* test. *P<0.05, **P<0.01, ***P<0.001.

Similarly, the percentage of normal slow waves was significantly reduced in the MetS+hyperglycaemia group at both anatomical sites during both preprandial and postprandial recordings (all P < 0.01, **Figure 4B**), indicating heightened susceptibility to rhythm instability. The hyperglycaemia and MetS+euglycaemia groups exhibited comparable numerical reductions, although these were not statistically significant (**Figure 4B**).

Waveform response area measurements demonstrated significant reductions in the MetS+hyperglycaemia group compared with controls at both the gastric body (preprandial: P < 0.001; postprandial: P < 0.05, **Figure 4C**) and the antrum (preprandial: P < 0.01; postprandial: P < 0.05, **Figure 4C**), suggesting reduced gastric motility reserve. Additionally, the hyperglycaemia group also showed reduced fasting waveform response areas at both the corpus (P < 0.05) and the antrum (P < 0.01) (**Figure 4C**). Overall, however, the waveform response area values in the MetS+hyperglycaemia group were numerically lower than those in the hyperglycaemia-alone group.

Regarding the dominant power ratio, the MetS+hyperglycaemia group showed significant decreases at both gastric sites during both preprandial and postprandial periods (all P < 0.05; **Figure 4D**), reflecting rhythm instability. The MetS+euglycaemia group demonstrated a reduced antral dominant power ratio (P < 0.05, **Figure 4D**), whilst the hyperglycaemia group showed non-significant downward trends.

Taken together, these EGG parameters demonstrate progressive autonomic impairment along the metabolic continuum, with the MetS+hyperglycaemia group exhibiting the most severe disturbances across all metrics. Notably, although the MetS+euglycaemia group showed limited statistically significant changes—likely due to the smaller sample size—the consistent numerical trends and the significant reductions in select antral parameters suggest that overweight/obesity, hypertension, and dyslipidaemia may contribute to gastric autonomic dysfunction beyond the effects of hyperglycaemia alone. These findings support the notion that cumulative MetS components collectively impair neurological regulation of gastrointestinal function.

## 4 Discussion

This integrated translational investigation provides evidence that neurological manifestations in MetS are associated with a multisystem metabolic burden driven by synergistic interactions amongst its components, rather than hyperglycaemia alone. The correspondence between rat models and human subgroups is summarised in **Supplementary Table S3**.

In a previous review, our group proposed that MetS may be more accurately termed metabolic dysfunction syndrome (MDS) (33). Notably, both the “Cardiovascular-Kidney-Metabolic (CKM) Syndrome” framework introduced by the American Heart Association (34) and the “systemic metabolic disorders” concept outlined by the European Atherosclerosis Society (35) share a common clinical phenotype rooted in metabolic dysfunction—irrespective of whether hyperglycaemia is present. The term MDS thus highlights the broader spectrum of metabolic impairments that often precede overt T2D, positioning other MetS components as upstream pathophysiological drivers (33). Importantly, neither of these authoritative statements addresses neuropathy as part of the disease spectrum (34, 35). Our findings therefore suggest that neurological assessments merit consideration in the clinical evaluation of metabolic disorders.

We interpret MetS-related neural injury within the broader spectrum of microvascular complications, analogous to retinopathic and nephropathic processes. Hyperglycaemia-mediated nerve damage involves well-established pathways—including polyol flux, hexosamine activation, advanced glycation end-product formation, mitochondrial dysfunction, and endoplasmic reticulum stress in Schwann cells and neurons (36). However, accumulating evidence underscores significant pathogenic contributions from dyslipidaemia, obesity, and hypertension to non-glycaemic neuropathies. Substantial causal evidence implicates dyslipidaemia as a direct neuropathic contributor: clinical trials have demonstrated that fenofibrate reduces diabetic retinopathy progression and stabilises non-proliferative retinopathy (37, 38), establishing triglyceride-mediated microvascular injury as a pathogenic mechanism. This parallels rodent data showing that dyslipidaemia alters nerve lipid composition and induces endoplasmic reticulum stress in ganglia (39, 40). Similarly, hypertension independently predicts the development of symmetrical polyneuropathy and is a significant risk factor for peripheral neuropathy in individuals with diabetes (41, 42), likely through angiotensin II-mediated reduction of endoneurial blood flow and endothelial dysfunction (43, 44)—mechanisms mirroring its established role in retinal and renal microvascular damage (45, 46). Regarding obesity, clinical investigations suggest that peripheral neuropathy is linked to obesity, which can result in reduced functional capacity (47). The causal role of obesity is further supported by evidence that weight-loss interventions, including bariatric surgery, can reverse neuropathy in affected individuals (48–50).

Insulin resistance (IR) serves as a unifying pathophysiological axis in MetS. Robust clinical evidence confirms its independent correlation with neuropathy severity, and the BARI 2D trial demonstrated that IR-improving strategies (using insulin sensitizers) reduce neuropathy incidence compared with insulin-replacement therapy (51). Recent studies have also suggested that increased insulin use may exacerbate IR, potentially increasing the risk of diabetic peripheral and autonomic neuropathy (52, 53). The mechanistic link between IR and neuronal damage warrants further investigation. Evidence from Drosophila indicates that ubiquitin C-terminal hydrolase L1 (UCHL1) deficiency induces both sensory neuropathy and IR (54). Moreover, individuals with MetS have been found to exhibit reduced levels of non-essential amino acids, and emerging evidence suggests that dysregulation of insulin-regulated serine and lipid metabolism contributes to peripheral neuropathy (55).

Although MetS diagnostic criteria do not currently include metabolic dysfunction-associated steatotic liver disease (MASLD), patients with MetS frequently exhibit concomitant MASLD, as also observed in our animal models (**Supplementary Table 4**; **Figure 2**). Clinical evidence suggests that MASLD increases the risk of microvascular complications even in the absence of hyperglycaemia or hypertension (56). Our rodent models corroborated this systemic vulnerability, demonstrating that hepatic steatosis severity correlates with neuropathy progression in normoglycaemic groups.

Currently, major clinical guidelines do not recommend routine neuropathy screening in non-T2D individuals with MetS components (57, 58). Consequently, nerve function assessments are rarely performed in patients with central adiposity, hypertension, or dyslipidaemia. Moreover, due to the limited availability of diagnostic tools in community settings, early detection of neuropathy remains challenging—particularly for visceral autonomic neuropathy (59). In our study, the consistency between rodent models and human data supports the vulnerability of peripheral nerves to non-glycaemic metabolic factors. Specifically, our human EMG data demonstrated that tibial and superficial peroneal nerve impairments were evident in the MetS+euglycaemia group, whereas peroneal nerve dysfunction was primarily driven by hyperglycaemia. This differential pattern suggests that sensory nerve fibres may be more susceptible to non-glycaemic metabolic insults than motor fibres—a finding that aligns with the length-dependent nature of metabolic neuropathies. Regarding autonomic nerves, we observed differential susceptibility amongst autonomic subsystems. Cardiac autonomic neuropathy was evident across all affected groups, including those without diabetes, as indicated by reduced HRV in humans. In contrast, gastrointestinal autonomic dysfunction—assessed by EGG—only reached statistical significance in the presence of hyperglycaemia, although the MetS+euglycaemia group showed consistent numerical trends. This organ-specific pattern may reflect the higher density of autonomic ganglia in the myocardium, differences in blood–tissue barrier permeability, or distinct neurotrophic factor requirements amongst tissues. The observation that EGG abnormalities were predominantly diabetes-associated suggests that cardiac autonomic dysfunction may serve as an earlier biomarker for autonomic neural injury in MetS. Furthermore, the progressive decline in HRV observed across human groups—from isolated hyperglycaemia to full MetS with hyperglycaemia—suggests the cumulative neurotoxic effects of individual MetS components.

To verify that the observed group differences were not attributable to demographic confounding, we performed multivariable linear regression for six key outcomes across all assessed modalities, adjusting for age and sex (**Supplementary Table S5**). After adjustment, the MetS + euglycaemia group remained independently associated with reduced right superficial peroneal SCV, RMSSD, and SDNN, whereas right tibial MCV and antral normal slow-wave percentage remained significantly reduced only in hyperglycaemia-containing groups. These adjusted analyses support the interpretation that non-glycaemic metabolic burden independently affects sensory and cardiac autonomic nerves, whilst motor and gastrointestinal autonomic function is more closely tied to hyperglycaemia—further supporting the organ-specific susceptibility pattern observed in the primary analyses.

Several limitations warrant consideration. First, oral glucose tolerance testing was not performed in the animal models, which would have provided a more comprehensive assessment of glucose homeostasis and insulin sensitivity beyond fasting glucose and insulin levels. Second, blood pressure was only monitored in hypertension-related models. Third, only male rats were used in the animal experiments to avoid the confounding effects of oestrous cycling on metabolic and neurological parameters; however, this limits the generalisability of the findings to female populations. Fourth, due to the cross-sectional design of our human study, the number of individuals with hyperglycaemia or MetS was smaller than that of normoglycaemic controls, making it difficult to achieve balanced group sizes. Fifth, some participants declined specific tests, resulting in incomplete data for certain modalities (e.g., some underwent EMG but not ECG or EGG). Sixth, the intriguing differential vulnerability between cardiac and gastric autonomic nerves merits further investigation through prospective studies. Seventh, detailed information on antihypertensive and glucose-lowering medication use was not collected in this community-based survey. Given that these medications may independently affect autonomic function, we cannot exclude the possibility of confounding. Eighth, the EGG subgroup analysis was limited by the small sample size in the MetS+euglycaemia group (n=25), which reduced statistical power to detect moderate effect sizes. This should be considered when interpreting the non-significant findings in this subgroup. Ninth, the multivariable regression analyses only adjusted for demographic covariates (age and sex) and did not account for all potential confounding factors.

## 5 Conclusion

In conclusion, this investigation suggests that neurological injury in MetS represents a distinct entity, separable from—or at least not solely attributable to—classic diabetic complications. The identification of significant neuropathy in normoglycaemic individuals with MetS, alongside the differential patterns observed across nerve subtypes, is consistent with a re-evaluation of current clinical approaches. We advocate for the incorporation of neurological assessments into routine management for patients with MetS to facilitate early diagnosis and potentially prevent the development of severe neuropathy.

## Data Availability

The raw data supporting the conclusions of this article will be made available by the authors, without undue reservation.
