# Supplementary material for: Neurological manifestations in metabolic dysfunction syndrome are associated with cumulative metabolic burden beyond hyperglycaemia: a translational study
Source: Front Endocrinol (Lausanne). 2026 Sep 28;17:1946996. doi: 10.3389/fendo.2026.1946996 (PMC13654758; doi:10.3389/fendo.2026.1946996)
Supplement: Supplementary file 1 [file Supplementaryfile1.docx]

**Supplementary Table S1.** Composition of experimental diets

| Diet | Model | Fat (%) | Carbohydrate (%) | Protein (%) | Additional components |
| --- | --- | --- | --- | --- | --- |
| 1010009 | Control | 12.79 | 66.67 | 20.54 | — |
| XTHF45 | Obesity | 47.18 | 33.29 | 19.54 | — |
| XT301 | Hyperlipidaemia | 31.33 | 49.03 | 19.64 | High fructose + cholesterol |
| XT109 | Atherogenic diet | 40.27 | 42.74 | 16.99 | 1.25% cholesterol + 0.5% sodium cholate |

**Supplementary Table S2**. Summary of the ten rat models used in the present study

| Full name | Abbreviation | Rat strain | Metabolic phenotype (Fig. 1) | Neuropathic phenotype (Fig. 1) |
| --- | --- | --- | --- | --- |
| Normal | - | Wistar | Normal body weight, BP, fasting glucose, insulin, and lipids; normal HOMA-IR | Normal HWL; normal SNCV |
| Hyperlipidaemia | - | Wistar | Elevated TG, TC, and LDL-C | HWL not significantly different from control; reduced SNCV |
| Obesity | - | Wistar | Increased body weight;hyperinsulinaemia and elevated HOMA-IR with normoglycaemia (insulin resistance) | HWL not significantly different from control; reduced SNCV |
| Hypertension | - | SHR | Systolic BP > 140 mmHg | Prolonged HWL; reduced SNCV |
| Hypertension + hyperlipidaemia | HBP+hyperlipidaemia | SHR | Elevated SBP with dyslipidaemia | Prolonged HWL; reduced SNCV |
| Type 2 diabetes + obesity | T2D + obesity | Wistar | Hyperglycaemia (>16.7 mmol/L); reduced fasting insulin with elevated HOMA-IR | Prolonged HWL; reduced SNCV |
| Type 2 diabetes + obesity + hyperlipidaemia | MetS1:DOL | Wistar | Hyperglycaemia with obesity and dyslipidaemia; insulin deficiency | Prolonged HWL; reduced SNCV |
| Hypertension + obesity + hyperlipidaemia | MetS2:HOL | SHR | Elevated SBP with obesity and dyslipidaemia | Prolonged HWL; reduced SNCV |
| Type 2 diabetes + hypertension + obesity | MetS3:DHO | SHR | Hyperglycaemia with hypertension and obesity | Prolonged HWL; reduced SNCV |
| Type 2 diabetes + hypertension + obesity + hyperlipidaemia | MetS4:DHOL | SHR | Hyperglycaemia with hypertension, obesity, and dyslipidaemia | Prolonged HWL; reduced SNCV |

Abbreviations: BP, blood pressure; HOMA-IR, homeostatic model assessment of insulin resistance; HWL, hindpaw withdrawal latency; LDL-C, low-density lipoprotein cholesterol; SNCV, Sciatic nerve conduction velocity; SBP, systolic blood pressure; T2D, type 2 diabetes; TC, total cholesterol; TG, triglycerides. T2D+obesity, type 2 diabetes + obesity;HBP+hyperlipidaemia, hypertension + hyperlipidaemia; MetS1:DOL, type 2 diabetes + obesity + hyperlipidaemia; MetS2:HOL, hypertension + obesity + hyperlipidaemia; MetS3:DHO, type 2 diabetes + hypertension + obesity; MetS4:DHOL, type 2 diabetes + hypertension + obesity + hyperlipidaemia. Neuropathic phenotypes are based on Fig. 1G (HWL) and Fig. 1H (SNCV); significance relative to control is indicated in Fig. 1.

**Supplementary Table S3.** Mapping of rat models to corresponding human subgroups

| Rat Models | Human Subgroup | Rationale |
| --- | --- | --- |
| Normal, Hyperlipidaemia, Obesity, Hypertension, HBP+Hyperlipidaemia | Euglycaemia | Non-diabetic (no hyperglycaemia); N/O/L/H are single‑component references, HL is multi‑component but still non‑diabetic |
| Type 2 diabetes+obesity | Hyperglycaemia (± obesity) | Hyperglycaemia present but only 2 metabolic components  (does not meet MS criteria) |
| MetS2:HOL | MetS+euglycaemia | Multi‑component (≥3) non‑diabetic MetS |
| MetS1:DOL,MetS3:DHO,MetS4:DHOL | MetS+hyperglycaemia | Multi‑component (≥3) MetS with hyperglycaemia |

Abbreviations: T2D+obesity, type 2 diabetes + obesity;HBP+hyperlipidaemia, hypertension + hyperlipidaemia; MetS1:DOL, type 2 diabetes + obesity + hyperlipidaemia; MetS2:HOL, hypertension + obesity + hyperlipidaemia; MetS3:DHO, type 2 diabetes + hypertension + obesity; MetS4:DHOL, type 2 diabetes + hypertension + obesity + hyperlipidaemia.

**Supplementary Table S4.** Hepatic enzyme levels in rat groups

| Group | ALT(U/L) (n=6) | AST(U/L) (n=6) |
| --- | --- | --- |
| Normal | 84.73±6.871 | 263.70±17.66 |
| Obesity | 42.40±6.638 | 209.07±34.31 |
| Hyperlipidemia | 53.67±7.185 | 156.50±13.11 |
| HBP | 192.33±8.716 | 418.20±21.38* |
| T2D+obesity | 87.03±7.148 | 186.60±28.60 |
| HBP+ hyperlipidemia | 179.00±19.38 | 409.00±8.194* |
| T2D+obesity+ hyperlipidemia (MS1:DOL) | 99.76±16.81 | 206.23±17.41 |
| HBP+obesity+ hyperlipidemia (MS2:HOL) | 214.67±17.67* | 414.00±24.71* |
| T2D+HBP+obesity (MS3:DHO) | 227.33±13.59* | 413.00±16.02* |
| T2D+HBP+obesity+ hyperlipidemia (MS4:DHOL) | 238.00±14.35* | 429.00±18.17* |

Data are expressed as mean±SEM. ALT, Alanine Aminotransferase, AST, Aspartate Aminotransferase，HBP, hypertension, T2D, type 2 diabetes, MS, metabolic syndrome. * Denotes P < 0.05 compared to the normal group.

**Supplementary Table S5**. Multivariable linear regression results for key nerve function outcomes.

| Outcome | Group vs. euglycamia | β (95% CI) | P value |
| --- | --- | --- | --- |
| Right superficial peroneal SCV (m/s) | Isolated hyperglycaemia | -0.33 (-1.51 to 0.84) | 0.576 |
|  | MetS + euglycaemia | -1.36 (-2.68 to -0.03) | 0.045 |
|  | MetS + hyperglycaemia | -1.28 (-2.28 to -0.27) | 0.013 |
| Right tibial MCV (m/s) | Isolated hyperglycaemia | -2.36 (-3.39 to -1.34) | <0.001 |
|  | MetS + euglycaemia | 0.36 (-0.78 to 1.49) | 0.537 |
|  | MetS + hyperglycaemia | -3.08 (-3.93 to -2.23) | <0.001 |
| RMSSD (ms) | Isolated hyperglycaemia | -2.69 (-5.19 to -0.20) | 0.034 |
|  | MetS + euglycaemia | -3.04 (-5.29 to -0.79) | 0.008 |
|  | MetS + hyperglycaemia | -2.43 (-4.28 to -0.57) | 0.01 |
| SDNN (ms) | Isolated hyperglycaemia | -2.15 (-4.11 to -0.18) | 0.033 |
|  | MetS + euglycaemia | -3.04 (-4.82 to -1.26) | 0.001 |
|  | MetS + hyperglycaemia | -2.12 (-3.58 to -0.67) | 0.004 |
| Antrum normal slow waves %, preprandial | Isolated hyperglycaemia | -0.76 (-3.76 to 2.24) | 0.618 |
|  | MetS + euglycaemia | -3.49 (-7.02 to 0.04) | 0.053 |
|  | MetS + hyperglycaemia | -4.35 (-6.97 to -1.72) | 0.001 |
| Antrum normal slow waves %, postprandial | Isolated hyperglycaemia | 1.01 (-2.28 to 4.31) | 0.546 |
|  | MetS + euglycaemia | -1.06 (-4.94 to 2.82) | 0.591 |
|  | MetS + hyperglycaemia | -4.14 (-7.02 to -1.26) | 0.005 |

Abbreviations: RMSSD, the root mean square of successive difference;SDNN, the standard deviation of normal-to-normal intervals;
