# Supplementary material for: Neurological manifestations in metabolic dysfunction syndrome are associated with cumulative metabolic burden beyond hyperglycaemia: a translational study
Source: Front Endocrinol (Lausanne). 2026 Sep 28;17:1946996. doi: 10.3389/fendo.2026.1946996 (PMC13654758; doi:10.3389/fendo.2026.1946996)
Supplement: Supplementary file 2 [file Supplementaryfile2.docx]

**Supplementary Figure 1**

**Supplementary Figure 1.** Aortic atherosclerosis assessment in MetS model rats. Hematoxylin and Eosin (H&E) staining of aortic tissues from all groups at 20 and 28 weeks of age. Magnification: ×200. Abbreviations: T2D+obesity, type 2 diabetes + obesity; MetS2:HOL, hypertension + obesity + hyperlipidaemia; MetS1:DOL, type 2 diabetes + obesity + hyperlipidaemia; MetS3:DHO, type 2 diabetes + hypertension + obesity; MetS4:DHOL, type 2 diabetes + hypertension + obesity + hyperlipidaemia.

**Supplementary Figure 2**

 **Supplementary figure 2. Hepatic histopathological alterations under optical microscopy of each group at 20 weeks of age.** Magnification 400×, Scale bar = 10 µm. MS, metabolic syndrome. N, normal. O, obesity. L, hyperlipidemia. H, HBP, Hypertension. D, T2D.
